# PSYCHOMETRIC PROPERTIES OF LOADED AND UNLOADED TIMED STAIR TESTS IN ASSESSING PEOPLE WITH KNEE OSTEOARTHRITIS

**DOI:** 10.2340/jrm.v58.46246

**Published:** 2026-08-03

**Authors:** Shamay S. M. NG, Siyue LI, Peiming CHEN, Eve LI, Carey K. L. LI, Hoi-Ying LO, Mimi M. Y. TSE, Cynthia Y. Y. LAI

**Affiliations:** 1 Department of Rehabilitation Sciences, The Hong Kong Polytechnic University, Hong Kong; 2 Research Centre for Chinese Medicine Innovation, The Hong Kong Polytechnic University, Hong Kong; 3 School of Nursing and Health Studies, Hong Kong Metropolitan University, Hong Kong, China

**Keywords:** osteoarthritis, knee, motor activity, physical functional performance, outcome assessment

## Abstract

**Objective:**

To compare timed stair test performance between individuals with knee osteoarthritis and healthy older adults, examine reliability under loaded and unloaded conditions, explore correlations with functional measures, and identify optimal cut-off times.

**Design:**

Cross-sectional study.

**Subjects/Patients:**

Sixty individuals with knee osteoarthritis and 30 healthy older adults.

**Methods:**

Participants completed unloaded and loaded timed stair test trials 7 days apart. Knee strength, balance, mobility, and self-reported outcomes were assessed.

**Results:**

Mean completion times for osteoarthritis participants were 23.91 s (unloaded) and 25.40 s (loaded). Intra-rater (ICC_3,1_ = 0.951–0.990 across raters and assessment days) and inter-rater (ICC_3,2_ = 0.999 [unloaded] to 1.000 [loaded]) reliabilities were excellent. Test–retest reliability was good for loaded (0.835) and excellent for unloaded (0.918) conditions. Times correlated negatively with affected side knee strength, balance, and confidence, and positively with pain severity, mobility tests, and daily activity difficulty. Optimal cut-offs were 18.67 s (unloaded) and 19.94 s (loaded), demonstrating sensitivities of 70.0% (unloaded) and 68.3% (loaded), a specificity of 86.7%, and area under the curve values of 0.78 (unloaded) and 0.77 (loaded).

**Conclusion:**

The timed stair test is a reliable, valid, and practical measure of functional performance in knee osteoarthritis, effectively distinguishing movement capability under loaded and unloaded conditions.

## LAY ABSTRACT

Climbing stairs is often a difficult daily task for people with knee joint wear and tear (osteoarthritis). This study investigated whether a specific timed stair-climbing test is a consistent and accurate way to measure mobility, both with and without carrying extra weight. We tested 60 people with knee osteoarthritis and 30 healthy older adults. The results showed that the stair test is highly consistent every time it is used. The time it took people to complete the test closely mirrored their actual leg strength, balance, and daily activity struggles. We also found specific time scores (around 18 to 20 s) that accurately separate people with knee problems from healthy adults. For doctors and physical therapists, this means the timed stair test is a quick, practical tool to evaluate a patient’s physical abilities and track their improvement during rehabilitation.

Knee osteoarthritis (KOA) is a degenerative condition of the knee joint characterized by the progressive loss of articular cartilage, leading to pain, stiffness, and mobility limitations (1). Globally, KOA affects more than 650 million people, with prevalence increasing markedly with age (2). Beyond pain and joint degeneration, KOA impairs lower-limb biomechanics through muscle weakness, reduced neuromuscular control, and joint instability, leading to progressive functional decline. Among daily tasks, stair negotiation is particularly challenging for individuals with KOA, as it requires greater knee range of motion, strength, and balance than level walking. Difficulty with stairs is an early, disabling limitation associated with poorer quality of life (3, 4). Consequently, stair-climbing performance directly reflects deficits in strength, balance, and pain tolerance, making it a clinically meaningful indicator of overall functional status (5).

To assess physical function in people with hip or knee osteoarthritis, the Osteoarthritis Research Society International (OARSI) recommends several performance-based tests to complement patient‑reported outcome measures, including the Timed Stair Test (TST) for stair negotiation (6). Together with the Chair Stand Test and the 40‑m Fast‑Paced Walk Test (FPWT), the TST forms part of the minimal core set recommended for both clinical and research use, underscoring the critical need for objective assessments of stair‑climbing ability. Traditional TST protocols typically require ascending and descending 4–5 steps and have been widely used in older adults and individuals with hip or knee osteoarthritis (7, 8). These tests are limited by their simplified task components, which may not adequately reflect the biomechanical complexity of stair negotiation encountered in daily life. To address this limitation, a more comprehensive version of the TST was developed that integrates multiple functional components, including rising from a chair, ascending and descending stairs, level walking, and returning to a seated position, performed with or without an external load. Previous studies have demonstrated adequate to excellent psychometric properties of the comprehensive TST in some populations. Specifically, the TST showed adequate reliability in individuals with obesity (intraclass correlation coefficient [ICC] = 0.88) (9), and excellent intra‑rater reliability (ICC = 0.985–0.991) along with acceptable concurrent validity among stroke survivors (10). Furthermore, stair‑focused performance tests have demonstrated good to excellent test–retest reliability (ICC = 0.83–0.95) and adequate validity in individuals with KOA (11–13), displaying strong associations with FPWT performance, quadriceps strength, balance measures, and the Western Ontario and McMaster Universities Osteoarthritis Index (WOMAC) scores (11).

Despite this evidence, a critical gap remains regarding the comprehensive TST’s psychometric properties in individuals with KOA. Specifically, its reliability, validity, and discriminative ability under both unloaded and loaded conditions – which accurately reflect the physical demands of real‑world stair negotiation, such as carrying groceries – remain unclear. Addressing this gap is essential to establish the comprehensive TST as a robust and clinically meaningful assessment tool. Therefore, this study aimed to: (*i*) compare TST performance between participants with KOA and healthy older adults; (*ii*) assess the intra‑rater, inter‑rater, and test–retest reliability of TST completion times under both loaded and unloaded conditions; (*iii*) examine concurrent validity with knee‑related functional assessments; and (*iv*) determine the optimal TST cut‑off values for differentiating stair‑climbing performance.

## Methods

### Study design

This cross-sectional study was carried out between October 2024 and June 2025 in a university-affiliated neurorehabilitation laboratory at the Hong Kong Polytechnic University, Hong Kong SAR, China. All participants were fully informed regarding the study’s procedures, and written informed consent was obtained from each participant prior to data collection. Ethical approval was granted by the University Ethics Committee of the local institution (HSEARS20240919003), and all procedures were conducted in accordance with the principles of the Declaration of Helsinki.

**Fig. 1:**
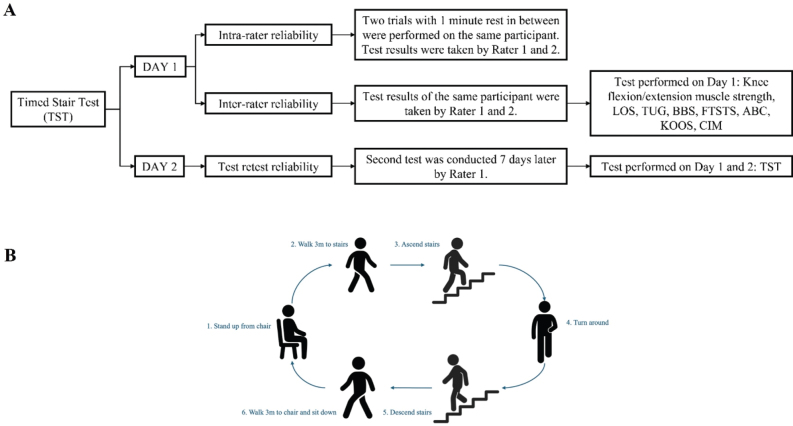
Methodology and setup of the Timed Stair Test (TST). (A) Overview of the testing procedures utilized to assess the intra-rater, inter-rater, and test–retest reliability across Day 1 and Day 2. (B) Schematic representation of the TST setup used for performance assessment, demonstrating the complete testing cycle.

All participants attended 2 testing sessions (Day 1 and Day 2) separated by a 1‑week interval (Fig. 1A). On Day 1, baseline assessments were performed, followed by reliability testing of TST. Participants underwent assessments of knee flexion and extension muscle strength, Limits of Stability (LOS), Berg Balance Scale (BBS), Timed Up‑and‑Go test (TUG), Five‑Times Sit‑to‑Stand Test (FTSTS), Activity‑specific Balance Confidence (ABC) scale, Knee injury and Osteoarthritis Outcome Score (KOOS), and Community Integration Measure (CIM). The TST was also administered on Day 1 for intra‑rater and inter‑rater reliability analyses. On Day 2, the TST was repeated to evaluate test–retest reliability. No additional clinical or functional measures were collected on this day.

Intra‑rater reliability was evaluated on Day 1 by having the same rater administer 2 TST trials, separated by a 1‑minute rest interval. Inter‑rater reliability was assessed on Day 1 by 2 independent raters who simultaneously timed each TST trial; the average of the 2 raters’ measurements was used for analysis. Test–retest reliability was determined by comparing TST completion times obtained on Day 1 and Day 2, which were separated by a 1‑week interval.

### Participants

A total of 60 community-dwelling individuals with KOA and 30 healthy older adults were recruited. Inclusion criteria were: (*i*) aged 55 years or older; (*ii*) a unilateral diagnosis of KOA confirmed by a qualified medical practitioner and supported by radiographic evidence; (*iii*) the ability to ascend and descend stairs independently, with or without handrail support; (*iv*) an Abbreviated Mental Test score of 7 or higher. Exclusion criteria were: (*i*) bilateral KOA; (*ii*) a history of knee surgery; (*iii*) presence of other musculoskeletal conditions causing pain or functional limitation greater than knee symptoms; and (*iv*) neurological or cardiovascular diseases that could affect physical performance or test safety. The same inclusion and exclusion criteria were applied to healthy older adults, with the exception of a knee osteoarthritis diagnosis.

### Sample size calculation

Previous research examining the Timed Stair Test (TST) reported excellent reliability (intraclass correlation coefficient [ICC]_2,1_ = 0.83; 95% confidence interval [CI] = 0.71–0.90) in adults with hip osteoarthritis and KOA (11). Based on this reference, and assuming a minimum acceptable ICC of 0.65, a sample size of 48 participants was calculated to provide 80% statistical power at a 0.05 significance level, using an online sample size calculator for reliability studies (14).

In addition, a strong correlation between TST and TUG performance was previously reported in individuals with KOA (*r* = 0.71; *p* < 0.001) (15). Based on this finding, a power analysis using G*Power version 3.1.9.7 (Franz Faul, University of Kiel, Kiel, Germany) indicated that a minimum of 13 participants would be required to detect a similar correlation in a 2‑tailed test with α = 0.05 and power = 0.80. To allow for more conservative assumptions (*r* = 0.35) and to ensure sufficient power for both reliability and validity analyses with multiple outcome measures, a final sample size of 60 participants was adopted for the KOA group.

### Outcome measures

*Timed Stair Test (TST).* The TST is a performance‑based assessment developed to measure higher‑level functional mobility (16). The test consists of 6 sequential tasks: (*i*) rising from a standard armless chair with back support, (*ii*) walking 3 m toward a staircase, (*iii*) ascending a 12‑step staircase, (*iv*) turning at the top of the stairs, (v) descending the staircase, and (*vi*) walking 3 m back to the chair and sitting down again (Fig. 1B). The staircase used for testing consisted of 12 steps, each measuring 13 cm in height and 24 cm in tread length (10). Participants completed the test at their self‑selected walking pace without the use of assistive devices. Handrail support (height: 90 cm) was allowed if required and its use was documented.

The total completion time was recorded in seconds using a stopwatch, with 2 independent raters measuring the performance simultaneously. Each participant completed 2 trials under loaded conditions, followed by 2 trials under unloaded conditions, with no practice trials. In the loaded condition, participants wore a weighted vest equivalent to 5% of their bodyweight. The vest load was adjusted using metal strips weighing 0.16 kg each to match individual bodyweight. For reliability analyses, the 2 trials performed under the same conditions were used as repeated measures. For intra‑rater reliability, the 2 trials recorded by the same rater were compared. For inter‑rater reliability, timing values recorded simultaneously by the 2 raters for the same trial were analysed, using the mean of the 2 raters as the final score. For test–retest reliability, TST completion times obtained on Day 1 and Day 2 were compared. Where applicable, the mean completion time of the 2 trials under each condition was used for subsequent analyses. The TST has shown high test–retest reliability (ICC_2,1_ = 0.83; 95% CI = 0.71–0.90) among community-dwelling individuals with hip osteoarthritis or KOA (11).

*Isometric knee flexion/extension muscle strength.* Bilateral isometric strength of the knee flexor and extensor muscles was evaluated using a Cybex dynamometer (Cybex International, Medway, MA, USA) (17). Participants were seated and stabilized with straps, with the hip flexed at 85–90° and the tested knee fixed at 90° of flexion. Participants performed 3 maximal voluntary isometric contractions for each muscle group, instructed to push or pull “as hard and as fast as possible” . A 1‑minute rest was provided between trials. The mean peak torque (Nm) of the 3 trials for each limb was used for analysis.

*Limits of Stability (LOS).* Dynamic balance and postural control were evaluated using the LOS test on a computerized dynamic posturography system (Bertec Corporation, Columbus, OH, USA). Participants stood barefoot on a force platform and used real-time visual feedback to shift their centre of pressure (COP) towards 8 directional targets. Primary outcome measures included reaction time (RT), movement velocity (MV), endpoint excursion (EE), maximum excursion (ME), and directional control (DC). Each participant completed 2 trials, with composite scores calculated as the mean performance across all 8 directions (18).

*Berg Balance Scale (BBS).* The BBS is a widely used tool for assessing functional balance in older adults. It consists of 14 functional tasks, each scored on a scale from 0 to 4, with a maximum possible score of 56. Higher scores indicate better balance performance (19).

*Timed Up and Go (TUG).* The TUG test is a widely used measure of functional mobility. During the assessment, participants were instructed to stand up from a chair, walk 3 m, turn, return to the chair, and sit down. Each participant completed 1 practice trial to become familiar with the procedure, followed by 2 timed trials. The mean completion time of the 2 trials was used for analysis (20).

*Five Times Sit to Stand (FTSTS).* The FTSTS is used to evaluate lower‑limb muscle strength and postural control in older adults. Participants were instructed to cross their arms over their chest and rise from an armless chair to a full standing position and return to sitting 5 times as quickly as possible without using their arms. One practice trial was provided, followed by 2 timed trials. The completion time was recorded, with longer times indicating poorer functional performance and a higher risk of falls (21).

*Activities-specific Balance Confidence (ABC) Scale.* The ABC Scale is a 16‑item self‑administered questionnaire that evaluates perceived balance confidence and fall risk during various indoor and outdoor activities. Each item is scored on a scale from 0 to 100, with higher scores indicating greater balance confidence and functional ability (22).

*Knee Injury and Osteoarthritis Outcome Score (KOOS).* The Knee Injury and Osteoarthritis Outcome Score (KOOS) is a knee‑specific patient‑reported outcome measure comprising 5 domains: pain, symptoms, activities of daily living (ADL), sport and recreation function, and knee‑related quality of life (QOL) (23). The questionnaire comprises 42 items, each rated on a 5‑point Likert scale ranging from 0 to 4. Domain scores are subsequently converted to a 0–100 scale, where lower values reflect greater symptom severity and poorer functional status.

*Community Integration Measure (CIM).* The CIM is a 10‑item self‑report questionnaire designed to assess an individual’s perceived level of community integration. Each item is scored on a 5‑point Likert scale, producing a total score between 10 and 50, with higher scores reflecting greater integration within the community (24).

### Statistical analysis

All statistical analyses were conducted using IBM SPSS Statistics version 28 (IBM Corp, Armonk, NY, USA). Descriptive statistics, including means and standard deviations (SDs) for interval data and medians, quartiles, and ranges for ordinal data, were computed to summarize participant demographics and outcome variables. The Kolmogorov–Smirnov test and Levene’s test were employed to assess normality and homogeneity of variance, respectively. All continuous TST completion times and interval-level outcomes (knee muscle strength, LOS parameters, TUG, FTSTS, BBS, and ABC scores) were approximately normally distributed; given the group sizes (*n* = 60 and *n* = 30), the sampling distributions of their means could also be reasonably assumed to be normal under the central limit theorem, and these variables were therefore analysed using parametric tests. In contrast, the ordinal and bounded KOOS subscale scores departed from normality; non-parametric tests were selected for these variables because they do not assume an underlying normal distribution and are more appropriate for ordinal and skewed data. For within‑group comparisons (Day 1 vs Day 2 and loaded vs unloaded conditions), paired‑samples *t*‑tests were applied to normally distributed data, whereas the Wilcoxon signed‑rank test was used for non‑normal data. Between‑group comparisons between participants with KOA and healthy older adults were conducted using independent‑samples *t*‑tests for parametric variables and Mann–Whitney *U* tests for non‑parametric variables.

Intraclass correlation coefficients (ICCs) were computed to determine the reliability of the measurements. The ICC_3,1_ model (two‑way mixed‑effects model, absolute agreement, single measurement) was used to assess intra‑rater and test–retest reliability, as repeated assessments were conducted by the same examiner (25), while the ICC_3,2_ model (two‑way mixed‑effects model, absolute agreement, mean of multiple measurements) was used to assess inter‑rater reliability, as data were collected simultaneously by 2 raters. ICC values were interpreted as follows: < 0.50 = poor, 0.50–0.75 = moderate, 0.75–0.90 = good, and > 0.90 = excellent reliability (25).

Associations between TST completion times and other outcome measures were examined using Pearson’s correlation coefficients (*r*) for normally distributed variables and Spearman’s rank correlation coefficients (ρ) for non‑normally distributed variables. To account for multiple comparisons across 8 primary outcomes, the Bonferroni correction was applied, with statistical significance set at *p* ≤ 0.006 (0.05/8). Correlation coefficients were interpreted as follows: ≤ 0.25 = little or no correlation, 0.26–0.50 = fair, 0.51–0.75 = moderate to good, and > 0.75 = good to excellent relationship strength (26).

Receiver operating characteristic (ROC) curve analyses were conducted to determine the discriminative ability of TST completion times under loaded and unloaded conditions in differentiating participants with KOA from healthy older adults (27). Optimal cut‑off values were identified using the Youden Index, defined as sensitivity + specificity − 1.

## Results

### TST performance and between-group comparisons

A total of 60 individuals with KOA and 30 healthy older adults were included in the final analysis. The KOA group had a significantly higher weight and body mass index than the healthy older adults, with no significant differences in age or height (Table I). Participants with KOA took significantly longer to complete the TST under both loaded and unloaded conditions compared with healthy older adults (*p* < 0.001) (Table II). Furthermore, both groups required significantly more time to complete the TST under the loaded condition than the unloaded condition (*p* < 0.001).

**Table I:** Demographics of the people with knee osteoarthritis (KOA, *n* = 60) and the healthy older adults (*n* = 30)

Characteristics	KOA (*n* = 60)	Healthy (*n* = 30)	*p*-value
Age, years, mean (SD)	66.43 (6.05)	68.10 (6.54)	0.234
Sex, M/F, *n*	18/42	7/23	
KOA duration, months, mean (SD)	64.57 (66.03)	N/A	N/A
Height, cm, mean (SD)	1.60 (0.09)	1.60 (0.09)	0.903
Weight, kg, mean (SD)	62.00 (9.82)	56.52 (7.50)	0.01*
BMI, mean (SD)	24.20 (3.22)	22.12 (2.22)	0.002*
Dominant side, left/right, *n*	58/2	27/3	0.193
KOA-affected side, *n*			
Left	27	N/A	N/A
Right	33	N/A	N/A
Mobility status, *n* (%)			
Walks without gait aids	57 (95%)	N/A	N/A
Walks with gait aids	3 (5%)	N/A	N/A
Number of falls in the past year, mean (SD)	0.58 (1.21)	N/A	N/A

* Significant difference at the *p* ≤ 0.05 level of confidence; Δ Test–retest = mean of the paired within‑rater differences between Day 1 and Day 2 (Rater 1 only, as Day 2 was assessed by Rater 1); Δ Inter‑rater = mean of the paired between‑rater differences (Rater 1−Rater 2) on Day 1, reported on the mean score used for analysis. Data were complete for all participants (KOA, *n* = 60; healthy older adults, *n* = 30); no data were missing for any variable. TST: Timed Stair Test; SD: standard deviation; R1: Rater 1; R2: Rater 2; N/A: not applicable.

**Table II:** Mean TST completion time of the people with knee osteoarthritis (KOA) and the healthy older adults

		KOA (*n* = 60)						Healthy (*n* = 30)		
		Day 1 (s, mean (SD))	Day 2 (s, mean (SD))	ΔTest–retest, Day 1−Day 2 (s, mean (SD))	ΔInter‑rater, R1−R2, Day 1 (s, mean (SD))	*p*-value (day 1 vs day 2)	*p*-value (unloaded vs loaded)	Day 1 (s, mean (SD))	*p*-value (unloaded vs loaded)	*p*-value (KOA vs healthy older adults)
Condition 1: unloaded										
Rater 1	Trial 1	24.21 (9.02)	23.34 (7.95)	0.87 (3.73)	—	0.140	< 0.001*	17.14 (3.99)	< 0.001*	< 0.001*
	Trial 2	23.60 (8.72)	23.14 (7.95)	0.46 (3.48)	—	0.859	< 0.001*	17.03 (4.05)	< 0.001*	< 0.001*
	Mean	23.91 (8.79)	23.24 (7.93)	0.67 (3.28)	−0.45 (0.30)	0.253	< 0.001*	17.09 (4.01)	< 0.001*	< 0.001*
Rater 2	Trial 1	24.60 (9.23)	N/A	N/A	—	N/A	< 0.001*	17.13 (4.08)	< 0.001*	< 0.001*
	Trial 2	23.74 (8.67)	N/A	N/A	—	N/A	< 0.001*	17.03 (4.04)	< 0.001*	< 0.001*
	Mean	24.36 (8.92)	N/A	N/A	—	N/A	< 0.001*	17.08 (4.05)	< 0.001*	< 0.001*
Condition 2: loaded										
Rater 1	Trial 1	25.64 (9.49)	25.50 (11.09)	0.14 (6.19)	—	0.291	< 0.001*	18.74 (4.17)	< 0.001*	< 0.001*
	Trial 2	25.16 (9.30)	25.16 (10.21)	0.00 (5.44)	—	0.726	< 0.001*	17.91 (3.43)	< 0.001*	< 0.001*
	Mean	25.40 (9.28)	25.33 (10.64)	0.07 (3.28)	−0.05 (0.29)	0.616	< 0.001*	18.33 (3.76)	< 0.001*	< 0.001*
Rater 2	Trial 1	25.69 (9.48)	N/A	N/A	—	N/A	< 0.001*	18.65 (4.15)	< 0.001*	< 0.001*
	Trial 2	25.21 (9.25)	N/A	N/A	—	N/A	< 0.001*	17.89 (3.33)	< 0.001*	< 0.001*
	Mean	25.45 (9.25)	N/A			N/A	< 0.001*	18.27 (3.69)	< 0.001*	< 0.001*

* Significant difference at the *p* ≤ 0.05 level of confidence; SD: standard deviation; N/A: not applicable.

### Reliability

The reliability analysis of TST demonstrated high consistency across all evaluated parameters for individuals with KOA (Table III). The TST showed excellent intra‑rater reliability under both the loaded and unloaded conditions. For the loaded condition, the intra-rater ICC_3,1_ values were 0.952 for Rater 1 and 0.951 for Rater 2 on Day 1, and 0.990 for Rater 1 on Day 2; corresponding values for the unloaded condition were 0.959 (Rater 1, Day 1), 0.968 (Rater 2, Day 1), and 0.990 (Rater 1, Day 2). Inter‑rater reliability was also excellent for the loaded (ICC_3,2_ = 1.000) and unloaded (ICC_3,2_ = 0.999) condition. Because the 2 raters timed each trial simultaneously with stopwatches, their recorded values were virtually identical, which explains the near-perfect inter-rater ICCs. Test–retest reliability ranged from good to excellent, with ICC_3__,1_ = 0.835 for the loaded condition and ICC_3,1_ = 0.918 for the unloaded condition.

**Table III:** Intra-rater, inter-rater, and test–retest reliability of the Timed Stair Test in people with knee osteoarthritis (KOA, *n* = 60)

Reliability type	Assessment phase	ICC model	Unloaded condition ICC (95% CI)	Loaded condition ICC (95% CI)	*p*-value
Intra-rater	Rater 1, Day 1	ICC_3,1_	0.959 (0.932–0.976)	0.952 (0.922–0.971)	< 0.001*
	Rater 1, Day 2	ICC_3,1_	0.990 (0.983–0.994)	0.990 (0.983–0.994)	< 0.001*
	Rater 2, Day 1	ICC_3,1_	0.968 (0.947–0.980)	0.951 (0.920–0.970)	< 0.001*
Inter-rater	Day 1 (Rater 1 vs Rater 2)	ICC_3,2_	0.999 (0.999–1.000)	1.000 (0.999–1.000)	< 0.001*
Test-retest	Rater 1 (Day 1 vs Day 2)	ICC_3,1_	0.918 (0.864–0.951)	0.835 (0.738–0.899)	< 0.001*

* Significant difference at the *p* ≤ 0.05 level of confidence; ICC: intraclass correlation coefficient; CI: confidence interval. All reliability analyses were performed within the KOA group (*n* = 60). Data were complete for all participants; no data were missing for any variable.

### Correlations between TST and other outcome variables

Under both unloaded and loaded conditions, affected‑side knee flexor strength and affected side extensor strength showed fair negative correlations with TST completion times (Table IV) (unloaded: *r* = –0.408 to –0.400; loaded: *r* = –0.410 to –0.397). The Pain and Knee‑related QOL subscales of the KOOS demonstrated fair negative correlations with TST times (unloaded: *r* = –0.407 to –0.355; loaded: *r* = –0.392 to –0.375). Scores on the BBS, total KOOS, the ADL and Sports/Recreation subscales of the KOOS, and ABC scale exhibited moderate‑to‑good negative correlations with TST completion times (unloaded: *r* = –0.657 to –0.522; loaded: *r* = –0.606 to –0.501). Conversely, TUG and FTSTS demonstrated moderate‑to‑good positive correlations (unloaded: *r* = 0.602–0.719; loaded: *r* = 0.557–0.718). No significant associations were observed with the remaining outcome measures.

**Table IV:** Correlations performance between Timed Stair Test (TST) with KOA-specific impairments in people with KOA (*n* = 60)

TST Rater 1	Unloaded		Loaded	
	*r*	*p*-value	*r*	*p*-value
Knee flexor strength				
Affected	–0.400	0.002*	–0.410	0.001*
Unaffected	–0.171	0.303	–0.177	0.180
Knee extensor strength				
Affected	–0.408	0.001*	–0.397	0.002*
Unaffected	–0.344	0.008	–0.342	0.008
BBS	–0.627	< 0.001*	–0.600	< 0.001*
TUG	0.719	< 0.001*	0.718	< 0.001*
CIM	–0.187	0.153	–0.209	0.109
5STS	0.602	< 0.001*	0.557	< 0.001*
KOOS				
Symptoms	–0.268	0.380	–0.238	0.067
Pain	–0.407	0.001*	–0.392	0.002*
Difficulty experienced in ADL	–0.530	< 0.001*	–0.501	< 0.001*
Sports and recreation	–0.583	< 0.001*	–0.575	< 0.001*
Knee-related QOL	–0.355	0.005*	–0.375	0.003*
Total	–0.522	< 0.001*	–0.505	< 0.001*
ABC	–0.657	< 0.001*	–0.606	< 0.001*
LOS				
RT	0.239	0.082	0.230	0.094
MV	–0.318	0.016	–0.355	0.007
EE	–0.420	0.001*	–0.434	< 0.001*
ME	–0.350	0.008	–0.361	0.006*
DC	–0.250	0.061	–0.259	0.051

Correlations are Pearson’s coefficients, and Spearman’s rho coefficients (*r*_s_).

* Significant correlation after Bonferroni adjustment at a *p*-value of 0.05/8 (*p* ≤ 0.006).

BBS: Berg Balance Scale; TUG: Timed Up and Go Test; CIM: Community Integration Measure – Chinese Version; 5STS: Five Times Sit to Stand Test; KOOS: Knee Injury and Osteoarthritis Outcome Score; ADL: activities of daily living; QOL: quality of life; ABC: Activity-specific Balance Confidence Scale; LOS: Limit of Stability Test; RT: reaction time; MV: movement velocity; EE: endpoint excursions; ME: maximum excursions; DC: directional control. All correlation analyses were performed within the KOA group (*n* = 60). Data were complete for all participants; no data were missing for any variable.

### Cut-off scores

**Fig. 2:**
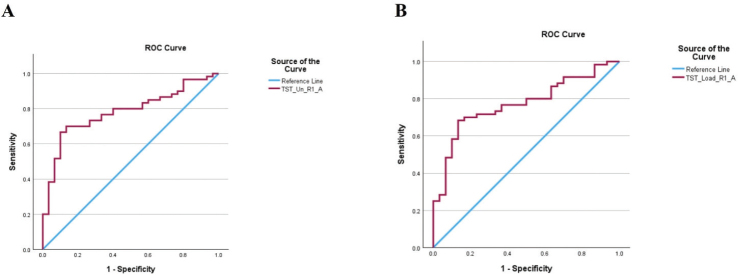
Receiver operating characteristic (ROC) curves illustrating the ability of the Timed Stair Test (TST) to differentiate individuals with knee osteoarthritis (KOA, *n* = 60) from healthy older adults (*n* = 30). (A) Diagnostic performance under the unloaded condition (AUC = 0.782, sensitivity = 70.0%, specificity = 86.7%). (B) Diagnostic performance under the loaded condition (AUC = 0.771, sensitivity = 68.3%, specificity = 86.7%). Data were complete for all participants; no data were missing for any variable.

ROC curve analyses identified optimal cut‑off values for TST completion times of 19.94 s under the loaded condition and 18.67 s under the unloaded condition. These cut‑off scores demonstrated moderate discriminative ability, with sensitivities ranging from 68.3% to 70.0%, a specificity of 86.7%, and an AUC ranging from 0.771 to 0.782 (Fig. 2).

## DISCUSSION

This study is the first to evaluate both intra‑rater and inter‑rater reliability and to determine optimal cut‑off values for TST in individuals with KOA. Individuals with KOA demonstrated significantly slower TST completion times than healthy older adults under both loaded and unloaded conditions, confirming impaired stair‑climbing performance associated with KOA. The TST showed excellent intra‑rater reliability, excellent inter‑rater reliability under loaded conditions, and good to excellent test–retest reliability, supporting its consistency across testing conditions. Fair to good correlations between TST completion times and knee muscle strength, balance, functional mobility, pain, and knee‑related quality of life further confirmed the concurrent validity of the TST. Additionally, optimal cut‑off values of approximately 18–20 s demonstrated fair discriminative ability to differentiate stair‑climbing performance between individuals with KOA and healthy older adults. Collectively, these findings support the TST as a sensitive, reliable, and clinically feasible measure of stair‑negotiation ability in individuals with KOA.

### TST performance

Participants with KOA demonstrated significantly longer TST completion times than healthy older adults, with completion times increasing proportionally when an external load was applied. Increased external loading has been shown to amplify knee joint moments and metabolic demand, leading to slower movement to maintain stability and reduce pain (28). Compared with previous findings in stroke rehabilitation, where people with stroke exhibited drastically prolonged completion times relative to healthy controls (10), the magnitude of impairment observed in our KOA cohort suggests a distinct, disease-specific profile of functional mobility limitation.

Stair climbing places substantial demands on knee extensor strength and controlled knee flexion, both of which are commonly compromised in KOA due to cartilage degeneration and osteophyte formation (29), resulting in pain‑related quadriceps inhibition, joint stiffness, and reduced range of motion (30, 31). These pathological changes limit force generation and shock absorption at the knee, resulting in compensatory movement strategies such as increased trunk forward lean, reduced ankle dorsiflexion, and prolonged sit‑to‑stand and stair‑negotiation phases, thereby lengthening TST completion time. The markedly longer TST times observed in the KOA group compared with healthy older adults under both conditions reflect these disease‑specific impairments in knee joint loading tolerance and neuromuscular control during stair ascent and descent.

A relative vest load of 5% bodyweight was selected to optimize the balance between participant safety and the sensitivity of the TST to KOA-related deficits. This approach, where relative loading accounts for individual differences in body mass and baseline strength, has been shown to successfully increase mechanical demands, specifically augmenting knee flexion moments in older adults, while remaining generally tolerable (10, 32). The observed increase in TST completion time under loaded conditions in the KOA group (6.3% increase) is consistent with the principle that external loading increases metabolic and biomechanical demands, often resulting in slower movement as an adaptive strategy to maintain stability and reduce pain. Furthermore, the significant increase in TST completion time observed under loaded conditions in healthy older adults supports the TST’s sensitivity to increased mechanical demands on the knee joint. In contrast, previous studies found that healthy older adults in a stroke study did not show a significant difference in TST completion time between unloaded and loaded conditions, suggesting that a 5% bodyweight load may not be sufficient to impact the stair-climbing speed of healthy participants significantly (33). This highlights that the TST under loaded conditions appears particularly effective in revealing subclinical mobility limitations or greater reliance on compensatory strategies when functional reserves are challenged, as evidenced by the KOA cohort.

### TST reliability

The TST demonstrated excellent intra-rater and inter-rater reliability among individuals with KOA under both loaded and unloaded conditions. These findings strongly align with OARSI recommendations, which recognize stair-negotiation tests as highly reliable performance-based measures (ICC > 0.85) for assessing physical function in individuals with hip or knee osteoarthritis (6). Furthermore, previous studies have similarly reported excellent test–retest reliability (ICCs > 0.90) for timed stair performance in patients with lower limb joint pathologies (34, 35).

The robust reliability observed in the present study can likely be attributed to the standardized testing protocol, clear verbal instructions, and the involvement of experienced raters. The strong test–retest reliability is further supported by the tightly controlled testing environment and the relatively short retest interval of 7 days. This interval is strategically brief enough to minimize true clinical changes in pain, strength, or functional status related to KOA progression, while simultaneously reducing cognitive biases such as recall or practice effects. Finally, conducting sequential assessments within an identical venue effectively controlled for extraneous environmental variables, specifically architectural stair dimensions and surface friction characteristics, which significantly contributed to the superior measurement stability.

### Correlations between TST and other outcome variables

Under both loaded and unloaded conditions, knee flexor strength on the affected side and bilateral knee extensor strength showed fair negative correlations with TST completion time. These results align with earlier studies highlighting quadriceps strength as a key contributor to stair‑climbing (15). Knee extensors determine power during stair negotiation (10), while knee flexors are vital for dynamic joint stability (36). In KOA, diminished strength and impaired eccentric control directly limit stair-climbing capacity (37). However, the observed fair correlations reflect that TST performance also integrates balance, coordination, and proprioceptive control.

LOS parameters (EE, ME, and MV) showed fair negative correlations with TST completion time, highlighting that dynamic postural shifts are crucial for stair climbing. The TST requires continuous, controlled displacement of the centre of gravity over a changing base of support. Conversely, the lack of correlation with RT and DC suggests that overall task duration is dominated by physical execution and momentum regulation rather than initial motor planning or strict directional control (10, 38).

A moderate‑to‑good inverse relationship was observed between BBS scores and TST completion time. This association is attributable to the shared construct of functional dynamic balance and similarities between several BBS items (e.g., postural transitions, turning) and TST subtasks. Higher BBS scores imply greater competence in managing equilibrium, directly translating to faster TST performance.

The TUG test showed moderate to good positive correlations with TST completion times. This expected association is due to identical core functional components shared by both assessments: rising, walking, turning, and sitting. The TST effectively acts as an augmented TUG test with increased mechanical and balance demands from stair ambulation. Thus, basic locomotor capacity measured by TUG is a prerequisite for better TST scores.

The FTSTS also showed moderate‑to‑strong positive associations with TST performance. The FTSTS primarily measures lower-limb strength and power, specifically the quadriceps’ capacity to repeatedly overcome gravity (39). Previous studies note that older adults with poorer FTSTS performance experience greater difficulty with stair‑climbing (40). Because quadriceps power is critical for vertical displacement, faster FTSTS performance reliably predicts the lower limb explosive power needed for efficient TST execution.

The ABC scale demonstrated moderate to good negative correlations with TST completion time. Low balance confidence reflects a high fear of falling, leading individuals to adopt overly cautious and slow movement strategies during the TST. This deliberate pacing to minimize perceived risk directly prolongs completion time.

Pain and knee‑related QOL subscales of the KOOS showed fair positive correlations with TST. Pain acts as a potent inhibitor of motor unit recruitment, limiting the maximal force required for rapid stair climbing (41). The ADL and Sport and Recreation subscales demonstrated moderate to good positive correlations, as these domains include high-level physical activities involving stairs. The Symptom subscale lacked significant correlation, likely because static symptoms influence maximal-effort tasks less than dynamic pain (42). Overall, total KOOS scores showed a moderate‑to‑strong inverse relationship with TST performance.

The CIM showed no significant correlation with TST under either condition. This lack of association stems from the fundamental difference in the constructs measured. The TST is an objective, performance-based measure of physical capacity. In contrast, the CIM captures subjective, self-reported perceptions of community participation (43), which are significantly mediated by psychosocial factors, environmental barriers, and personal motivation that extend far beyond physical stair-climbing speed.

### TST cut-off

The TST demonstrated fair discriminative ability in differentiating stair‑climbing performance between individuals with KOA and healthy older adults. Optimal cut‑off values were identified at 18.67 s for the unloaded condition and 19.94 s for the loaded condition, with corresponding AUC values of 0.782 and 0.771, respectively (44). These cut‑off scores indicate an approximately 77–78% probability of correctly distinguishing stair‑climbing performance between the 2 groups, supporting the clinical utility of the TST as a screening tool for KOA‑related functional impairment.

### Limitations

Several limitations should be acknowledged. First, the affected knee was not necessarily the dominant limb, which may have contributed to variability in TST performance. Second, only individuals with unilateral KOA were included, and KOA severity was not stratified, limiting the generalizability of the findings to individuals with bilateral involvement or more advanced disease, in whom stair‑climbing performance and reliability may differ. Third, the cognitive capacity required for task comprehension and execution was not formally assessed and may have influenced performance. Fourth, handrail use during the TST was neither standardized nor controlled, which could have affected completion times and movement strategies. Fifth, the study focused on completion time alone; movement quality and compensatory strategies were not evaluated, limiting insight into biomechanical adaptations or potential safety risks during stair negotiation. In addition, inter-rater reliability was established using only 2 experienced raters who timed each trial simultaneously; the resulting near-perfect agreement may partly reflect the involvement of the same 2 professionals, and generalization to a broader pool of raters with differing experience warrants further study. This does not diminish the demonstrated reliability of the test but should be considered when interpreting the inter-rater estimates. Finally, the sample included a higher proportion of female participants, which may influence the generalizability of the results given known sex‑related differences in KOA prevalence, pain perception, and muscle strength. Notwithstanding these limitations, because the TST requires only a standard staircase, a chair, and a stopwatch, and because its administration does not depend on culturally or geographically specific factors, we anticipate that the test and the reported cut‑off values are broadly applicable across clinical and community settings worldwide; nonetheless, local validation may be advisable where staircase dimensions differ substantially from those used here (12 steps; 13 cm rise, 24 cm tread). Future studies should examine the influence of disease severity, bilateral involvement, sex differences, cognitive factors, and movement strategies on stair‑climbing performance in individuals with KOA.

### Conclusion

The Timed Stair Test is a reliable and valid measure for assessing stair‑climbing ability and functional performance in individuals with knee osteoarthritis. In addition to demonstrating stable reliability and meaningful associations with knee strength, balance, functional mobility, pain, and knee‑related quality of life, the TST effectively differentiated individuals with knee osteoarthritis from healthy older adults. As a brief and straightforward test requiring minimal equipment, the TST is well suited for use in routine clinical and rehabilitation settings.
